# Editorial for the Special Issue on Carbon Based Electronic Devices

**DOI:** 10.3390/mi10120856

**Published:** 2019-12-06

**Authors:** Alberto Tagliaferro, Costas Charitidis

**Affiliations:** 1 Department of Applied Science and Technology, Politecnico Torino, Corso Ducadegli Abruzzi, 24, 10129 Torino TO, Italy; 2 Research Lab of Advanced, Composite, Nanomaterials and Nanotechnology, School of Chemical Engineering, National Technical University of Athens, 9 Heroon Polytechniou str., Zographou, Athens GR-15780, Greece

For more than 50 years, silicon has dominated the electronics industry. However, due to resources limitations, viable alternatives are considered and investigated. Among all alternative elements, carbon is the predominant element for a number of reasons; last but not least the fact that it can be obtained from waste. Whereas the physical properties of graphite and diamond have been investigated for many years, the potential for electronic applications of other allotropes of carbon (fullerenes, carbon nanotubes, carbon nanofibres, carbon films, carbon balls and beads, carbon fibres, etc), has only been appreciated relatively recently. Carbon-based materials offer a number of exciting possibilities for new applications of electronic devices, due to their unique thermal and electrical properties. However, the success of carbon-based electronics depends on the rapid progress of the fabrication, doping and manipulation techniques.

The present Special issue has a twofold structure: on one side review papers dealing with the most developed fields; on the other innovative research papers that report new exciting results. 

A wide spectra of carbon materials and a wide range of applications are described in the present issue. As per material type, papers deal with graphene and graphene-oxide [1,2,3,4], carbon nanotubes [2,5,6] and with other forms of carbon, such as porous carbon [7] and nanofibers [8,9]. A plethora of devices are witnessing the versatility of carbon materials: supercapacitors [1,9], non-volatile memories [8], pressure sensors [2,7], field-effect transistors [10], white-light photosensors [3], cold cathode electron emitters [5], gas and humidity detectors [6,11], MEMS and NEMS [12], carbon based inks for 3D microfluidic MEMS [13], transparent conductive electrodes [4].

Dywily et al. [1] describe the production of nanometal decorated graphene oxide anchored on PANI and its performance in supercapactors, achieving specific capacitance values up to 227.2 F/g; a value that favorably compares with other literature data involving graphene based systems. Bondavalli et al. [8] focus on the fabrication of Resistive Random Access Memory (ReRAM) on flexible substrates based on oxidized carbon nanofibres (CNFs) showing that two different resistance states (ON, OFF) reversibly switchable can be obtained. Caradonna et al. [2] discuss the use of various carbon nanofillers to promote piezoresistivity in polymers by means of laser scribing treatment able to produce conductive tracks in an otherwise low conductive material. Porous carbon electrodes and their interesting piezoresistive properties are discussed by Dai et al. [7]. An innovative (faster and cheaper) method to produce liquid-metal electrodes for graphene field-effect transistors is discussed by Melcher et al. [10]. The use of a new technique (active-screen plasma) to functionalize and decorate carbon nanofibres with metals for supercapacitor applications is presented by Li et al. [9]. A method aimed to overcome the limitation of standard approaches in preparing graphene-based photosensors is discussed and detailed by Tu et al. [3]. Kim et al. [5] focused their work on the fabrication of stable CNT cold emitter using an aging technique. The last research paper is presented by Song et al. [6] and focuses on the ability of a two-nanotube sensor to selectively detect NO and NO_2_.

The remaining papers of the issue are reviews aimed to provide to the reader an overview of several fields of interest, ranging from NEMS and MEMS [12] to functionalized carbon materials for electronic devices [14], from carbon-based humidity sensors [11] to graphene-based transparent conductive electrodes [4]. Finally, O’Mahony et al. [13] reviewed the rheological issues to be tackled in addictive manufacturing when using carbon-based inks for lab-on-a-chip applications.
